# Access to medicines, the Unified Health System, and intersectional injustices

**DOI:** 10.11606/s1518-8787.2024058005986

**Published:** 2024-07-22

**Authors:** Elba Marina Miotto Mujica, João Luiz Bastos, Alexandra Crispim Boing

**Affiliations:** I Universidade Federal de Santa Catarina Programa de Pós-Graduação em Saúde Coletiva Florianópolis SC Brasil Universidade Federal de Santa Catarina. Programa de Pós-Graduação em Saúde Coletiva. Florianópolis, SC, Brasil; II Simon Fraser University Faculty of Health Sciences Burnaby BC Canada Simon Fraser University. Faculty of Health Sciences. Burnaby, BC, Canada

**Keywords:** Accessibility to Health Services, Unified Health System, Intersectional Framework

## Abstract

**OBJECTIVE:**

To estimate the prevalence of general and public access to prescription drugs in the Brazilian population aged 15 or older in 2019, and to identify inequities in access, according to intersections of gender, color/race, socioeconomic level, and territory.

**METHODS:**

We analyzed data from the 2019 National Health Survey with respondents aged 15 years or older who had been prescribed a medication in a healthcare service in the two weeks prior to the interview (n = 19,819). The outcome variable was access to medicines, subdivided into general access (public, private and mixed), public access (via the Unified Health System - SUS) for those treated by the SUS, and public access (via the SUS) for those not treated by the SUS. The study’s independent variables were used to represent axes of marginalization: gender, color/race, socioeconomic level, and territory. The prevalence of general and public access in the different groups analyzed was calculated and the association of the outcomes with the aforementioned axes was estimated with odds ratios (OR) using logistic regression models.

**RESULTS:**

There was a high prevalence of general access (84.9%), when all sources of access were considered, favoring more privileged segments of the population, such as men, white, and those of high socioeconomic status. When only the medicines prescribed in the SUS were considered, there was a low prevalence (30.4% access) that otherwise benefited marginalized population segments, such as women, black, and people from low socioeconomic backgrounds.

**CONCLUSIONS:**

Access to medicines through the SUS proves to be an instrument for combating intersectional inequities, lending credence to the idea that the SUS is an efficient public policy for promoting social justice.

## INTRODUCTION

Access to medicines is fundamental to safeguarding the right to health, and has been recognized as an essential human right. This principle was included in the targets of the Millennium Development Goals and remains a central element in the 2030 Agenda for sustainable development, established by the United Nations (UN)^1^. On the global stage, injustices in access to medicines continue to be evident. Despite the efforts of the World Health Organization (WHO) to achieve the goal of universal health coverage, which includes access to medicines, there are still significant variations in access to medicines between countries. Medicines are completely absent from primary care in approximately 30% of 25 countries surveyed by the WHO^4^.

In Brazil, the Brazilian Health Reform movement and the subsequent establishment of the Unified Health System (SUS) transformed access to medicines. Initially limited to product availability, the perspective shifted strategically within the National Health Policy, emphasizing integrality. The National Medicines Policy (PNM) was approved based on the need to provide Pharmaceutical Assistance (AF) as a guiding policy for the formulation of sectoral policies. Referrals from the 1^st^ National Conference on Medicines and Pharmaceutical Services in 2003 resulted in the approval of the National Policy on Pharmaceutical Services (PNAF), through a resolution of the National Health Council. These policies established free access to essential medicines as a right for Brazilian citizens and made pharmaceutical services a public health policy in the country^5^. However, there are still different forms of organization and financing for access to medicines in the country: provision by the public health network (through the SUS), by the private sector through health plans, or by direct payment and mixed financing through the Popular Pharmacy Program^8^. In other universal health systems, such as those in the United Kingdom, Australia and Canada, co-payment is the main form of access to medicines, where part of the cost of the medicine is subsidized by the health system and the other part comes from direct payment by the user. Free access only occurs in specific situations and varies according to age and income or is based on specific health needs^9^.

Brazilian studies carried out since the 2000s have shown an increase in the prevalence of access to medicines, when all sources of access are considered. However, there are still significant inequities in access according to socioeconomic status and place of residence, as well as other axes of marginalization^10^. The studies carried out so far show that access to free medicines favors poorer people^13^, those with less schooling and those of black color/race^12^. In addition, studies have sought to assess specific populations, limiting themselves to the analysis of a single axis of marginalization and disregarding the interrelationships between multiple systems of oppression^17,18^. In health inequities research, an intersectionality approach has been highlighted. Conceived mainly in the wake of the U.S. black feminist movement of the 1980s, intersectionality has been used to explain how the experiences of individuals and groups are shaped by the intertwining and overlapping of multiple axes of marginalization and oppression, such as gender, race, and class, producing complex forms of oppression for some and privilege for others^19^.

Population-based studies on inequities in access to medicines are still scarce in Brazil^11,14^. This is concerning, particularly after 2016, when fiscal austerity measures were adopted and health policies were subjected to major underfunding, both of which have implications for social injustices and the worsening of health indicators^22^. In this scenario, an intersectional perspective can be valuable for identifying population segments lying at the intersections of multiple axes of oppression^25,26^ and for expanding knowledge about inequities in access to medicines in Brazil. In addition, there is a lack of population studies on the subject, both at national and international levels, using an intersectional perspective. With this in mind, the aim of the present study was to estimate the prevalence of general access and public access to prescription drugs in the Brazilian population aged 15 and over in 2019 and to identify inequities in access, according to the intersections of gender, color/race, socioeconomic status, and territory.

## METHODS

This study analyzed data from the 2019 National Health Survey (PNS), carried out by the Brazilian Institute of Geography and Statistics (IBGE) in partnership with the Ministry of Health (MS). The sampling frame included three stages of selection: Primary Sampling Units, represented by census tracts or a set of tracts, obtained from the IBGE Master Sample; households, selected by simple random sampling; and residents aged 15 or older, selected by simple random sampling, based on the list of residents obtained at the time of the interview^27,28^. In all, the PNS fieldworkers visited 108,525 households throughout Brazil and carried out 94,114 interviews, with a non-response rate of 6.4%. The questionnaire consisted of 26 modules, divided into three sections, which applied to the households, all residents, and a selected resident within each household. The first two sections were answered by a resident aged 18 or older who could provide information on the socio-economic and health conditions of all residents. Data collection took place between August 2019 and March 2020. The data obtained from the 2019 PNS are available on the IBGE website. The 2019 PNS was approved by the National Research Ethics Committee (Conep), under protocol no. 3.529.376^27,28^.

The analytical sample included only respondents aged 15 or older, living in private households in Brazil, who sought health care and were attended to in the two weeks prior to the interview, with the prescription of some medication during that visit. The outcome variable of this study was access to medicines. Three outcomes were assessed according to the source of the medication and the origin of care, which resulted in the prescription of the medication: general access, which considered all sources of medication (i.e., public, private and mixed) and origins of care (private care and SUS); public access, which considered only the SUS as a source of the medication, subdivided into those who obtained care from the SUS and those who did not.

To measure general access, we used the question: “Were you able to get the medicines you were prescribed?”. The possible answers were: “Yes, all”; “Yes, some”; and “No, none”. Access to medicines was characterized as a dichotomous variable, with the first response option being considered total access and the others, lack of total access. Public access for those served or not by the SUS was estimated using the question: “Were any of the medicines obtained from a public health service?”. In the same way, the response option “Yes, all” was considered total access and the response options “Yes, some” and “No, none”, lack of access. To determine the origin of the care provided, we used the question: “Was the care provided by the SUS?”, whose response options were “Yes”, “No”, and “Don’t know/Don’t remember”.

The independent variables were used to represent specific axes of marginalization. Gender, categorized dichotomously as “Male” or “Female”; color/race, collected according to the standard categories proposed by the IBGE, dichotomized as “White” and “Black”, with the “Black” and “Brown” categories included in the latter. Due to the small sample size, the “Yellow” and “Indigenous” categories were not considered in this study; socioeconomic level, operationalized by schooling, classified as “Low” (eight years of study or less) and “High” (more than eight years of study); and territory, operationalized by the macro-region of residence in the country (north, northeast, southeast, south, and midwest). Eight groups formed by the intersections of gender, color/race, and socioeconomic status were analyzed: white men of high socioeconomic status; white men of low socioeconomic status; black men of high socioeconomic status; black men of low socioeconomic status; white women of high socioeconomic status; white women of low socioeconomic status; black women of high socioeconomic status; and black women of low socioeconomic status. Subsequently, each intersectional group was analyzed according to the macro-region of residence.

A descriptive analysis of the sample was carried out, where the relative frequencies of the outcomes were estimated, accompanied by their 95% confidence intervals (95%CI), according to the axes of marginalization and intersectional groups. To test the association between the outcomes and the independent variables, three logistic regression models were run. Model 1 estimated the effect of each of the explanatory variables on the outcomes. Model 2 included adjustment between the variables gender, color/race, socioeconomic status, and territory. Model 3 was adjusted for intersectional groups and territory. The estimates of the regression coefficients, represented as odds ratios (OR), were also calculated with their 95%CI. Statistical analyses were carried out using Stata software, version 15.1, taking into account the weights and the complex sampling structure.

## RESULTS

The analytic sample consisted of 19,819 respondents aged 15 years or older. To analyze general access, data from all these respondents were taken into consideration. More than half had received care from the SUS, of which around 80% provided valid information on the outcome. On the other hand, less than half of the respondents had not been treated by the SUS and, among these, more than 80% provided valid information on access to medicines. In other words, for the last two outcomes, there were approximately 20% and 14%, respectively, of interviewees who answered the question about whether they were able to obtain the prescribed medicines but did not answer whether any of them were obtained from public health services.

Table 1 shows the description of the sample for the three outcomes, which was mostly made up of women living in the Southeast region. For the general access outcome, participants of black color/race and high socioeconomic status and, in the intersectional group, white women of high socioeconomic status were the most frequent participants. In the case of public access for those served by the SUS, most respondents were people of black color/race, low socioeconomic status and from the intersectional group of black women of low socioeconomic status. In the case of public access for those not served by the SUS, most respondents were white with a high socioeconomic status and at the intersection between white women and high socioeconomic status.

**Table 1:** Description of the sample according to gender, color/race, socioeconomic status, territory, intersectional group, and access to prescription drugs. National Health Survey, Brazil, 2019.

Variable	General access	Public access for SUS patients	Public access for those not covered by the SUS
		
	n (%)	n (%)	n (%)
Gender			
Man	6,985 (35.8)	3,526 (35.2)	2,261 (36.5)
Woman	12,834 (64.2)	6,463 (64.8)	4,024 (63.5)
Color/race			
White	7,851 (47.4)	3,252 (40.4)	3,290 (59.1)
Black	11,968 (52.6)	6,737 (59.6)	2,995 (40.9)
Socioeconomic status			
High	10,160 (52.6)	4,015 (41.0)	4,471 (71.0)
Low	9,659 (47.4)	5,974 (59.0)	1,814 (29.0)
Region			
South	2,685 (15.6)	1,274 (15.8)	917 (14.3)
Southeast	4,783 (47.2)	2,037 (42.4)	1,883 (54.0)
Midwest	2,220 (7.0)	970 (6.8)	839 (7.2)
Northeast	6,617 (23.9)	3,672 (27.7)	1,811 (19.7)
North	3,514 (6.3)	2,036 (7.3)	835 (4.8)
Intersectional group			
Men, white, high socioeconomic status	1,574 (10.0)	446 (6.2)	877 (15.9)
Men, white, low socioeconomic status	1,249 (7.2)	733 (8.3)	294 (5.4)
Men, black, high socioeconomic status	1,770 (8.1)	778 (7.0)	686 (9.6)
Men, black, low socioeconomic status	2,392 (10.5)	1,569 (13.7)	404 (5.6)
Women, white, high socioeconomic status	2,932 (17.7)	904 (11.6)	1,604 (27.5)
Women, white, low socioeconomic status	2,096 (12.6)	1,169 (14.3)	515 (10.3)
Women, black, high socioeconomic status	3,884 (16.8)	1,887 (16.3)	1,304 (18.0)
Women, black, low socioeconomic status	3,922 (17.1)	2,503 (22.6)	601 (7.7)
Access to prescription medicines			
No	3,139 (15.1)	6,906 (69.6)	6,124 (97.0)
Yes	16,680 (84.9)	3,083 (30.4)	161 (3.0)
Total	19,819	9,989	6,285

SUS: Unified Health System; CI: confidence interval.

Source: Elaborate by the authors based on data analysis from PNS, 2019.

The prevalence of general access found in this study was 84.9% (Table 2). When only access to medicines in the SUS was considered, with prescriptions originating in the system itself, the prevalence observed was 30.4%. The prevalence of public access among those whose prescriptions originated outside the SUS was 3.0%. Men, participants who reported being white, of high socioeconomic status, and living in the South had a higher prevalence of general access. When analyzing access to medicines in the public sector for prescriptions originating in the SUS, higher prevalence rates were observed among men, respondents of black color/race, respondents of low socioeconomic status, and residents of the southeast region. Regarding public access to medicines for prescriptions originating outside the SUS, the prevalence among men and women was similar, while higher prevalence rates were observed among respondents of black color/race, respondents with low socioeconomic status, and residents of the northeast region.

**Table 2:** Prevalence of general (public, private, and mixed) and public access (via SUS) to prescription drugs among respondents treated or not by the SUS, according to gender, color/race, socioeconomic status, territory, and intersectional group. National Health Survey, Brazil, 2019.

Variable	Prevalence of general access	Prevalence of public access to SUS services	Prevalence of public access for those not covered by the SUS
		
	% (95%CI)	% (95%CI)	% (95%CI)
Gender			
Man	86.8 (85.5–88.0)	30.7 (28.2–33.3)	3.0 (2.1–4.5)
Woman	83.8 (82.7–84.8)	30.2 (28.3–32.2)	3.0 (2.2–4.0)
Color/race			
White	86.6 (85.2–87.8)	29.5 (26.9–32.1)	2.5 (1.8–3.4)
Black	83.3 (82.1–84.5)	31.0 (29.0–33.0)	3.8 (2.7–5.4)
Socioeconomic status			
High	86.6 (85.4–87.7)	28.6 (26.3–31.1)	2.3 (1.6–3.1)
Low	83.0 (81.7–84.2)	31.6 (29.5–33.7)	4.8 (3.3–7.1)
Region			
South	86.1 (84.1–87.9)	31.6 (28.2–35.3)	3.1 (1.8–5.3)
Southeast	85.7 (84.1–87.2)	33.6 (30.6–36.7)	2.8 (1.9–3.9)
Midwest	85.5 (83.3–87.5)	26.3 (22.6–30.3)	1.8 (0.9–3.4)
Northeast	83.3 (81.8–84.7)	26.5 (24.4–28.6)	4.2 (2.4–7.2)
North	80.5 (78.1–82.7)	27.7 (24.8–30.9)	2.5 (1.4–4.5)
Intersectional group			
Men, white, high socioeconomic status	90.2 (87.7–92.2)	30.8 (23.8–38.8)	2.0 (1.0–4.0)
Men, white, low socioeconomic status	85.0 (81.8–87.7)	30.6 (25.7–36.1)	4.0 (1.8–8.9)
Men, black, high socioeconomic status	87.6 (84.9–89.9)	28.3 (23.5–33.6)	3.0 (1.3–6.6)
Men, black, low socioeconomic status	84.1 (81.7–86.3)	31.9 (28.4–35.6)	5.1 (2.1–12.2)
Women, white, high socioeconomic status	87.0 (84.9–88.9)	26.8 (22.3–31.8)	1.4 (0.9–2.2)
Women, white, low socioeconomic status	84.0 (81.6–86.2)	30.4 (26.4–34.6)	5.2 (3.2–8.5)
Women, black, high socioeconomic status	83.5 (81.5–85.4)	29.3 (26.2–32.6)	3.5 (2.0–6.0)
Women, black, low socioeconomic status	80.6 (78.5–82.6)	32.5 (29.1–36.0)	4.7 (2.3–9.1)
Total	84.9 (84.0–85.7)	30.4 (28.8–32.0)	3.0 (2.3–3.9)

SUS: Unified Health System.

Source: Elaborate by the authors based on data analysis from PNS, 2019.

White men of high socioeconomic status had the highest prevalence of general access, while black women of low socioeconomic status had the highest prevalence of public access among those treated at the SUS. The highest prevalence of public access among those treated outside the SUS was observed among white women of low socioeconomic status. The prevalence of the outcomes among each intersectional group, stratified by macro-region, was also analyzed. However, the estimates found were accompanied by low precision, as observed by the wide confidence intervals^a^.

Based on the analysis of the logistic regression models, Table 3 shows that the OR for prevalence of general access was lower for women, black respondents, those with low socioeconomic status (Models 1 and 2) and residents of the northern region (Models 1, 2, and 3). Both in the bivariate analysis (Model 1) and in the adjusted analysis (Model 3), all intersectional groups, except for black men of high socioeconomic status, had lower ORs for prevalence of general access compared to white men of high socioeconomic status.

**Table 3:** Odds ratios from logistic regression models for general access (public, private, and mixed) to prescription drugs according to exposure variables. National Health Survey, Brazil, 2019.

Variable	Model 1	Model 2	Model 3
		
	OR (95%CI)	OR (95%CI)	OR (95%CI)
Gender			
Man	1.00	1.00	-
Woman	0.79 (0.70–0.89)	0.78 (0.69–0.89)	-
Color/race			
White	1.00	1.00	-
Black	0.78 (0.68–0.89)	0.83 (0.72–0.96)	-
Socioeconomic status			
High	1.00	1.00	-
Low	0.75 (0.66–0.86)	0.77 (0.68–0.87)	-
Region			
South	1.00	1.00	1.00
Southeast	0.97 (0.79–1.19)	0.98 (0.80–1.21)	0.98 (0.80–1.20)
Midwest	0.95 (0.76–1.20)	1.01 (0.79–1.29)	1.01 (0.79–1.29)
Northeast	0.81 (0.67–0.98)	0.89 (0.73–1.09)	0.89 (0.73–1.09)
North	0.67 (0.54–0.83)	0.73 (0.58–0.92)	0.73 (0.58–0.92)
Intersectional group			
Men, white, high socioeconomic status	1.00	-	1.00
Men, white, low socioeconomic status	0.62 (0.44–0.87)	-	0.62 (0.44–0.87)
Men, black, high socioeconomic status	0.77 (0.55–1.08)	-	0.80 (0.57–1.14)
Men, black, low socioeconomic status	0.58 (0.43–0.79)	-	0.60 (0.44–0.83)
Women, white, high socioeconomic status	0.73 (0.55–0.98)	-	0.73 (0.55–0.98)
Women, white, low socioeconomic status	0.57 (0.42–0.77)	-	0.58 (0.43–0.78)
Women, black, high socioeconomic status	0.55 (0.42–0.73)	-	0.58 (0.44–0.77)
Women, black, low socioeconomic status	0.45 (0.34–0.60)	-	0.48 (0.35–0.64)

OR: Odds ratio; CI: confidence interval.

Source: Elaborate by the authors based on data analysis from PNS, 2019.

Regarding public access to the SUS (Table 4), interviewees from lower socioeconomic backgrounds had a higher OR for the prevalence of the outcome when compared to those from higher socioeconomic backgrounds (Model 2). Bivariate analysis indicated that residents of the northeast region had a lower OR for the prevalence of this type of access compared to residents of the south (Model 1). In the adjusted analysis, there was also a lower OR for this type of access among residents of the midwest and north regions (Models 2 and 3). Regarding the analysis of intersectional groups, no intersectional inequities were observed for this outcome in any of the models.

**Table 4:** Odds ratios from logistic regression models for public access (via SUS) to medicines prescribed in the SUS according to exposure variables. National Health Survey, Brazil, 2019.

Variable	Model 1	Model 2	Model 3
		
	OR (95%CI)	OR (95%CI)	OR (95%CI)
Gender			
Man	1.00	1.00	-
Woman	0.98 (0.84–1.13)	0.99 (0.85–1.15)	-
Color/race			
White	1.00	1.00	-
Black	1.07 (0.92–1.26)	1.17 (0.99–1.38)	-
Socioeconomic status			
High	1.00	1.00	-
Low	1.15 (0.99–1.33)	1.17 (1.01–1.36)	-
Territory			
South	1.00	1.00	1.00
South East	1.09 (0.88–1.35)	1.07 (0.86–1.34)	1.08 (0.87–1.34)
Midwest	0.77 (0.59–1.00)	0.73 (0.56–0.96)	0.73 (0.56–0.96)
North East	0.78 (0.64–0.95)	0.72 (0.59–0.89)	0.72 (0.59–0.89)
North	0.83 (0.66–1.04)	0.78 (0.61–0.99)	0.78 (0.61–0.99)
Intersectional group			
Men, white, high socioeconomic status	1.00	-	1.00
Men, white, low socioeconomic status	0.99 (0.65–1.52)	-	1.03 (0.68–1.58)
Men, black, high socioeconomic status	0.88 (0.58–1.36)	-	0.96 (0.63–1.48)
Men, black, low socioeconomic status	1.05 (0.71–1.55)	-	1.19 (0.80–1.75)
Women, white, high socioeconomic status	0.82 (0.54–1.25)	-	0.83 (0.54–1.26)
Women, white, low socioeconomic status	0.98 (0.65–1.48)	-	1.01 (0.67–1.52)
Women, black, high socioeconomic status	0.93 (0.63–1.37)	-	1.05 (0.71–1.53)
Women, black, low socioeconomic status	1.08 (0.73–1.60)	-	1.22 (0.83–1.81)

OR: Odds ratio; SUS: Unified Health System; CI: confidence interval.

Source: Elaborate by the authors based on data analysis from PNS, 2019.

Regarding public access for those treated outside the SUS (Table 5), a higher OR for prevalence of the outcome was also found among respondents from low socioeconomic backgrounds, when compared to those from high socioeconomic backgrounds (Models 1 and 2). In both the bivariate (Model 1) and the adjusted analysis (Model 3), the intersectional group of white women of low socioeconomic status had higher ORs for the prevalence of this type of access compared to white men of high socioeconomic status. However, these findings were accompanied by wide confidence intervals, due to the small sample size in relation to the other outcomes. A fourth model - not shown in the tables - tested the interaction between region and intersectional groups for the three outcomes. However, no significant interaction was identified.

**Table 5:** Odds ratios from logistic regression models for public access (via SUS) to medicines prescribed outside the SUS according to exposure variables. National Health Survey, Brazil, 2019.

Variable	Model 1	Model 2	Model 3
		
	OR (95%CI)	OR (95%CI)	OR (95%CI)
Gender			
Man	1.00	1.00	-
Woman	0.98 (0.62–1.55)	1.01 (0.63–1.62)	-
Color/race			
White	1.00	1.00	-
Black	1.57 (0.98–2.50)	1.51 (0.89–2.56)	-
Socioeconomic status			
High	1.00	1.00	-
Low	2.18 (1.29–3.67)	2.10 (1.24–3.56)	-
Region			
South	1.00	1.00	1.00
Southeast	0.88 (0.46–1.70)	0.81 (0.43–1.52)	0.81 (0.43–1.54)
Midwest	0.56 (0.24–1.33)	0.48 (0.20–1.14)	0.49 (0.20–1.16)
Northeast	1.35 (0.61–3.01)	1.07 (0.46–2.49)	1.09 (0.47–2.52)
North	0.79 (0.35–1.79)	0.60 (0.25–1.41)	0.62 (0.26–1.45)
Intersectional group			
Men, white, high socioeconomic status	1.00	-	1.00
Men, white, low socioeconomic status	2.03 (0.68–6.08)	-	2.04 (0.68–6.12)
Men, black, high socioeconomic status	1.50 (0.51–4.44)	-	1.50 (0.49–4.57)
Men, black, low socioeconomic status	2.63 (0.82–8.49)	-	2.63 (0.89–7.75)
Women, white, high socioeconomic status	0.68 (0.31–1.50)	-	0.69 (0.31–1.51)
Women, white, low socioeconomic status	2.67 (1.12–6.36)	-	2.64 (1.12–6.25)
Women, black, high socioeconomic status	1.74 (0.71–4.27)	-	1.77 (0.71–4.41)
Women, black, low socioeconomic status	2.37 (0.84–6.67)	-	2.37 (0.81–6.98)

OR: Odds ratio; SUS: Unified Health System; CI: confidence interval.

Source: Elaborate by the authors based on data analysis from PNS, 2019.

## DISCUSSION

Echoing other population-based studies, the prevalence of general access to prescription drugs was high when considering any type of source (public, private, and mixed)^12,14^. There was a higher prevalence of general access among more privileged population segments, such as white men of high socioeconomic status. When considering only access to medicines in the public system from prescriptions originating in the system itself, the prevalence is low but the scenario is reversed with a higher prevalence of access among people of black color/race and low socioeconomic status. There was even a higher prevalence of public access for black women from low socioeconomic backgrounds. These findings suggest that the SUS is an important source of access for those who are unable to obtain medicines outside the public system^29^. The co-payment modality, which is adopted by other universal health systems as the main way of obtaining medicines, is considered a hindrance to access. Higher co-payments are associated with a reduction in the volume of medicines dispensed, especially among the poorest, which leads to discontinuation of treatment, compromising the effectiveness of the healthcare provided^9^. Our findings corroborate other studies which indicate that the free provision of medicines reduces inequities in access^11,13,14,15,16,24^, also pointing out that access to medicines through the SUS is an important instrument for combating intersectional inequities.

Despite this, the low prevalence of access identified for obtaining prescription drugs from the SUS is concerning. In an analysis of data obtained from the 2008 National Household Sample Survey, Boing et al.^11^ identified a prevalence of public access of 45.3%, while in the 2013 PNS, the percentage identified was 31.6%^23^. As for the territory, represented by the country’s macro-regions, the analysis of the 2019 PNS showed that regional inequities in public access to medicines persist. Drummond et al.^14^, when analyzing the 2013 PNS, also identified a higher prevalence of access to prescription drugs in more developed regions with a higher population density. These findings indicate that, despite the progress made with the implementation of medicines and AF policies in the country, the provision of medicines by the public health system still remains a major challenge^30^.

In addition, the simultaneous presence of a universal public system in Brazil and a growing process of privatization of access to healthcare, further intensified by public underfunding of healthcare, are also worrying^31^. In this study, a significant portion of the population who did not have their needs met by the SUS tried to obtain the prescribed medicines through the public health system. For them, the prevalence of access to prescribed medicines was even lower. In addition to the well-known weaknesses in the structuring of pharmaceutical services in the country, such as the availability of medicines in SUS pharmacies^12^, there is the lack of knowledge among prescribers in the private sector of the essential medicines lists, reference lists that should guide the supply, prescription and dispensing of medicines in the SUS, and the lack of adherence to these lists on the part of prescribers in the public sector. When observing the demands for access to medicines through the courts in different Brazilian states, it can be seen that the majority of prescriptions originate outside the SUS and in approximately 75% of cases there is a therapeutic alternative to the prescribed medicine available through the SUS^34,35^.

Unlike general access, public access was not marked by significant intersectional inequities. In the models tested for public access to the SUS, no significant differences were identified between the intersectional groups examined. Access gaps between intersectional groups for general access (90.2% to 80.6%) are greater when compared to gaps in public access for those treated by the SUS (32.5% to 26.8%). This suggests that the differences in access observed between population segments are minimized when the medication is prescribed and obtained from the public health system. Monteiro et al.^36^, when evaluating the Generic Medicines Policy, also found that there was no statistically significant difference in the use of generic medicines in the population of the city of São Paulo according to age, sex, and schooling.

The intersectional analysis used helped identify the most invisible population groups. In addition, it was possible to observe that, while women had approximately 20% lower OR of general access, white women of high socioeconomic status had 27% lower OR and black women of low socioeconomic status had 55% lower OR when compared to white men of high socioeconomic status. The study by Katrein et al.^17^, when analyzing the prevalence of access to medicines for the treatment of chronic diseases, had already indicated a situation of greater vulnerability among those with a greater number of diseases and those who are poorer.

Although there are important challenges in using an intersectional perspective in epidemiological studies^37^, this approach stands out as the strength of this study. There are no other studies in the literature following an intersectionality perspective that are comparable to this one. The studies carried out so far, which have demonstrated the existence of inequalities in access to medicines in Brazil^17,18,24^, have assumed that variables, such as gender, color/race, and schooling are independent from one another.

Recent reviews have shown that the variables that characterize social positions can be operationalized in different ways^25,26^. In this study, as in most of those analyzed in the aforementioned reviews, intersectionality was operationalized using an exclusively intercategorical approach, based on the combination of categories of interest. However, comparisons between groups alone can provide a limited view of health inequities. In addition to disregarding intracategorical heterogeneity, this type of analysis focuses on social categories at the individual level, without considering the effect of broader social contexts^38^.

In addition, one of the limitations of the study was that it only included people who had been seen by a health service in the last two weeks prior to the interview. Considering that access to prescription drugs is directly related to access to health care, people who do not reach health services are excluded. It was observed that black people and those from lower socioeconomic backgrounds are more likely to report difficulty in accessing health services^39^. Furthermore, a possible memory bias can be assumed due to the recall period and self-report on the outcomes analyzed. However, a study comparing different recall periods identified little bias in the prevalence rates observed, and it is recommended that 14 days be used as the recall period to allow comparisons between studies^40^.

We conclude that the prevalence of general access to prescription drugs is higher for population segments with higher social status, while public access, which is still very small, favors those with lower social status, even when considering the intersection of multiple axes of marginalization. We therefore suggest that the SUS is a powerful means to promote social justice in access to medicines.

## Footnotes

a Supplementary Material available at: https://doi.org/10.7910/DVN/I0CL2L
