# Supplementary material for: Access to medicines, the Unified Health System, and intersectional injustices
Source: Rev Saude Publica. 2024 Jul 22;58:34. doi: 10.11606/s1518-8787.2024058005986 (PMC11319036; doi:10.11606/s1518-8787.2024058005986)
Supplement: Translation

# Translation: Acesso a medicamentos, o Sistema Único de Saúde e as injustiças interseccionais

**Authors:** Elba Marina Miotto Mujica, João Luiz Bastos, Alexandra Crispim Boing

**Affiliations:** I Brasil Universidade Federal de Santa Catarina. Programa de Pós-Graduação em Saúde Coletiva. Florianópolis, SC, Brasil; II Canada Simon Fraser University. Faculty of Health Sciences. Burnaby, BC, Canada

**Keywords:** Acessibilidade aos Serviços de Saúde, Sistema Único de Saúde, Enquadramento Interseccional

## Abstract

**OBJETIVO:**

Estimar as prevalências de acesso geral e público a medicamentos prescritos na população brasileira com 15 anos ou mais de idade em 2019 e identificar iniquidades de acesso, conforme intersecções de gênero, cor/raça, nível socioeconômico e território.

**MÉTODOS:**

Foram analisados dados da Pesquisa Nacional de Saúde de 2019 com respondentes de 15 anos ou mais que tiveram prescrição de algum medicamento em atendimento de saúde realizado nas duas semanas anteriores à entrevista (n = 19.819). A variável de desfecho foi o acesso a medicamentos, subdividido em acesso geral (público, privado e misto), acesso público (via Sistema Único de Saúde – SUS) dos atendidos no SUS e acesso público (via SUS) dos não atendidos no SUS. As variáveis independentes do estudo foram utilizadas para representar eixos de marginalização: gênero, cor/raça, nível socioeconômico e território. Foram calculadas as prevalências de acesso geral e acesso público nos diferentes grupos analisados e a associação dos desfechos com os eixos mencionados foi estimada com *odds ratios* (OR) por meio de modelos de regressão logística.

**RESULTADOS:**

Foi observada alta prevalência de acesso geral (84,9%), quando consideradas todas as fontes de obtenção, favorecendo segmentos populacionais de maior privilégio, como homens, brancos e de alto nível socioeconômico. Quando considerada apenas a obtenção no SUS dos medicamentos prescritos no próprio sistema, verificou-se uma baixa prevalência (30,4% de acesso), invertendo o acesso em benefício dos segmentos populacionais multiplamente marginalizados, como mulheres negras de baixo nível socioeconômico.

**CONCLUSÕES:**

O acesso a medicamentos por meio do SUS demonstra ser um instrumento de combate às iniquidades interseccionais, evidenciando que o SUS é uma política pública eficiente na promoção da justiça social.

## INTRODUÇÃO

O acesso a medicamentos é fundamental para a garantia do direito à saúde e tem sido reconhecido como um direito humano essencial. Esse princípio foi incluído nas metas dos Objetivos de Desenvolvimento do Milênio e permanece um elemento central na Agenda 2030 para o desenvolvimento sustentável, estabelecida pela Organização das Nações Unidas (ONU)^1^. No cenário mundial, as injustiças no acesso a medicamentos continuam a ser evidentes. Apesar dos esforços da Organização Mundial da Saúde (OMS) para o alcance da meta da cobertura universal de saúde, ainda se observam variações expressivas no acesso a medicamentos entre os países. Em aproximadamente 30% de 25 países pesquisados pela OMS, os medicamentos estão completamente ausentes no âmbito da atenção primária^4^.

No contexto brasileiro, com o movimento da Reforma Sanitária Brasileira e a consequente criação do Sistema Único de Saúde (SUS), o acesso a medicamentos passou de uma perspectiva limitada à disponibilidade do produto para um enfoque estratégico dentro da Política Nacional de Saúde na perspectiva da integralidade. A Política Nacional de Medicamentos (PNM) foi aprovada a partir da necessidade de garantir a Assistência Farmacêutica (AF) como política norteadora para formulação de políticas setoriais. Encaminhamentos da 1ª Conferência Nacional de Medicamentos e Assistência Farmacêutica de 2003 resultaram na aprovação da Política Nacional de Assistência Farmacêutica (Pnaf), por meio de uma resolução do Conselho Nacional de Saúde. Essas políticas estabeleceram o acesso gratuito a medicamentos essenciais como direito dos cidadãos brasileiros e efetivaram a AF como política pública de saúde^5^. Contudo, ainda existem diferentes formas de organização e financiamento para o acesso a medicamentos no país: fornecimento pela rede pública de saúde (por meio do SUS), pelo setor privado, através dos planos de saúde ou por pagamento direto e financiamento misto por meio do Programa Farmácia Popular^8^. Em outros sistemas universais de saúde, como os do Reino Unido, da Austrália e do Canadá, o copagamento é adotado como principal forma de acesso a medicamentos, em que parte do custo é subsidiado pelo sistema de saúde e a outra parte advém do desembolso direto do usuário. O acesso gratuito ocorre apenas em situações específicas e varia conforme a idade e a renda ou é baseado em necessidades de saúde específicas^9^.

Estudos brasileiros, realizados a partir dos anos 2000, evidenciaram um aumento na prevalência de acesso a medicamentos, quando consideradas todas as fontes de acesso. No entanto, ainda são identificadas importantes iniquidades de acesso entre segmentos da população, conforme condição socioeconômica e região de residência, além de outros eixos de marginalização^10^. Os estudos até então realizados evidenciam que o acesso a medicamentos de forma gratuita favorece pessoas mais pobres^13^, de menor escolaridade e de cor/raça preta^12^. Ademais, observam-se estudos que buscaram avaliar populações específicas, limitando-se à análise de um único eixo de marginalização e desconsiderando a inter-relação entre múltiplos sistemas de opressão^17,18^. Em pesquisas sobre iniquidades em saúde, tem-se destacado a abordagem da interseccionalidade. Concebida principalmente no bojo do movimento feminista negro dos anos 1980 nos Estados Unidos, a interseccionalidade tem sido acionada para explicar como as experiências de indivíduos e grupos são moldadas pelo entrelaçamento e pela sobreposição de múltiplos eixos de marginalização e opressão, como gênero, raça e classe, produzindo formas complexas de opressão para uns e de privilégio para outros^19^.

Estudos de base populacional sobre iniquidades no acesso a medicamentos ainda são escassos no Brasil^11,14^. Isso se torna ainda mais preocupante ao se considerar o contexto do país após 2016, com a adoção de medidas de austeridade fiscal e a consequente intensificação do subfinanciamento das políticas de saúde, que tem como implicações o agravamento das injustiças sociais e a piora de indicadores de saúde^22^. Nesse cenário, uma perspectiva interseccional pode ser valiosa para identificar segmentos populacionais que são multiplamente marginalizados^25,26^e para ampliar o conhecimento acerca das iniquidades no acesso aos medicamentos no Brasil. Além disso, destaca-se a ausência de estudos populacionais sobre o tema, tanto nacionais quanto internacionais, utilizando uma perspectiva interseccional. Com isso, o objetivo deste estudo foi de estimar as prevalências de acesso geral e acesso público a medicamentos prescritos na população brasileira com 15 anos ou mais de idade em 2019 e identificar iniquidades no acesso, conforme as intersecções de gênero, cor/raça, nível socioeconômico e território.

## MÉTODOS

No presente estudo, foram analisados dados da Pesquisa Nacional de Saúde (PNS) de 2019, realizada pelo Instituto Brasileiro de Geografia e Estatística (IBGE) em parceria com o Ministério da Saúde (MS). O plano amostral empregado foi por conglomerado, em três estágios de seleção: unidade primária de amostragem, representada pelos setores censitários ou conjunto de setores, obtida da Amostra Mestra do IBGE; domicílio, selecionado por amostragem aleatória simples; e morador com 15 anos ou mais de idade, selecionado por amostragem aleatória simples, com base na lista de moradores obtida no momento da entrevista^27,28^. Ao todo, os agentes de coleta da PNS visitaram 108.525 domicílios em todo o território brasileiro, tendo realizado 94.114 entrevistas, com taxa de não resposta de 6,4%. O questionário foi composto por 26 módulos, divididos em três seções: domiciliar, de todos os moradores e do morador selecionado. As duas primeiras seções foram respondidas por um morador de idade igual ou superior a 18 anos, que sabia informar sobre a situação socioeconômica e de saúde dos demais coabitantes. A coleta de dados ocorreu entre agosto de 2019 e março de 2020. Os dados obtidos na PNS 2019 são de domínio público, disponibilizados no *website* do IBGE. A PNS 2019 foi aprovada pela Comissão Nacional de Ética em Pesquisa (Conep), sob parecer nº 3.529.376^27,28^.

Na análise, foram incluídos apenas os respondentes de 15 anos ou mais, residentes em domicílios particulares do território brasileiro, que procuraram atendimento de saúde e foram atendidos nas duas semanas anteriores à entrevista, com a prescrição de algum medicamento nesse atendimento. A variável de desfecho deste estudo foi o acesso a medicamentos, considerando-se a obtenção dos medicamentos como medida de acesso. Foram avaliados três desfechos de acordo com a fonte de obtenção do medicamento e a origem do atendimento, que resultou na prescrição do medicamento: acesso geral, em que se considerou todas as fontes de obtenção (público, privado e misto) e origens do atendimento (rede privada e SUS); acesso público, na qual se considerou apenas a rede pública como fonte de obtenção do medicamento, subdivido entre aqueles que tiveram o atendimento realizado no SUS e os que não foram atendidos no SUS.

Para mensurar o acesso geral, foi utilizada a pergunta: “Conseguiu obter os medicamentos receitados?”. As possíveis respostas eram: “Sim, todos”; “Sim, alguns”; e “Não, nenhum”. Caracterizou-se o acesso a medicamentos como uma variável dicotômica, sendo a primeira opção de resposta considerada como acesso total e as demais, ausência de acesso total. O acesso público daqueles atendidos ou não no SUS foi estimado por meio da pergunta: “Algum dos medicamentos foi obtido em serviço público de saúde?”. Da mesma forma, a resposta “Sim, todos” foi considerada como acesso total e as respostas “Sim, alguns” e “Não, nenhum”, ausência de acesso. Para diferenciar a origem do atendimento realizado, foram utilizadas as respostas à pergunta: “O atendimento foi feito pelo SUS?”, cujas respostas eram “Sim”, “Não” e “Não sabe/Não lembra”.

As variáveis independentes foram utilizadas para representar eixos de marginalização específicos. Gênero, categorizado de forma dicotômica em “Homem” ou “Mulher”; cor/raça, coletada de acordo com as categorias padronizadas pelo IBGE, sendo dicotomizada em “Branca” e “Negra”, com as categorias “Preta” e “Parda” incluídas nessa última. Devido ao pequeno tamanho amostral, as categorias “Amarela” e “Indígena” não foram consideradas neste estudo; nível socioeconômico, operacionalizado pela escolaridade, classificado em “Baixo” (oito anos de estudo ou menos) e “Alto” (mais de oito anos de estudo); e território, operacionalizado pela macrorregião de residência no país (Norte, Nordeste, Sudeste, Sul e Centro-Oeste). Foram analisados oito grupos formados pelas intersecções de gênero, cor/raça e nível socioeconômico: homens brancos de alto nível socioeconômico; homens brancos de baixo nível socioeconômico; homens negros de alto nível socioeconômico; homens negros de baixo nível socioeconômico; mulheres brancas de alto nível socioeconômico; mulheres brancas de baixo nível socioeconômico; mulheres negras de alto nível socioeconômico; e mulheres negras de baixo nível socioeconômico. Posteriormente, cada grupo interseccional foi analisado segundo a macrorregião da residência.

Foi realizada a análise descritiva da amostra, tendo sido apresentadas as frequências relativas dos desfechos, acompanhadas de seus intervalos de confiança de 95% (IC95%), nas diferentes categorias dos eixos de marginalização e nos grupos interseccionais. Para testar a associação dos desfechos com as demais variáveis, foram analisados três modelos de regressão logística. No Modelo 1, foi estimado o efeito de cada uma das variáveis explicativas sobre os desfechos. O Modelo 2 incluiu o ajuste entre as variáveis gênero, cor/raça, nível socioeconômico e território. O Modelo 3 foi ajustado para os grupos interseccionais e o território. As estimativas dos coeficientes de regressão, representadas como *odds ratio* (OR), também foram calculadas com seus IC95%. As análises estatísticas foram realizadas no *software* Stata, versão 15.1, considerando os pesos e a estrutura amostral complexa.

## RESULTADOS

Foram considerados 19.819 entrevistados com idade maior ou igual a 15 anos. Para análise do acesso geral, as respostas de todos esses respondentes foram apreciadas. Do total, mais da metade teve atendimento realizado pelo SUS, sendo que, desses, cerca de 80% apresentaram informação válida quanto ao desfecho. Por sua vez, menos da metade dos respondentes não realizaram atendimento pelo SUS e, desses, mais de 80% apresentaram informação válida sobre o acesso a medicamentos. Ou seja, para os dois últimos desfechos, houve aproximadamente 20% e 14%, respectivamente, de entrevistados que responderam à pergunta sobre se conseguiram obter os medicamentos receitados, mas não responderam se algum deles foi obtido em serviço público de saúde.

A Tabela 1 apresenta a descrição da amostra para os três desfechos, que foi composta majoritariamente por mulheres residentes da região Sudeste. Para o desfecho de acesso geral, participantes de cor/raça negra, de alto nível socioeconômico e, do grupo interseccional, mulheres brancas de alto nível socioeconômico representaram a maioria dos participantes. No caso do acesso público dos atendidos no SUS, a maioria dos respondentes foi composta por pessoas de cor/raça negra, de baixo nível socioeconômico e do grupo interseccional de mulheres negras de baixo nível socioeconômico. Para o acesso público dos não atendidos no SUS, a maioria dos respondentes foi de pessoas de cor/raça branca, de alto nível socioeconômico e, do grupo interseccional, de mulheres brancas de alto nível socioeconômico.

**Tabela 1:** Descrição da amostra de acordo com gênero, cor/raça, nível socioeconômico, território, grupo interseccional e acesso aos medicamentos prescritos. Pesquisa Nacional de Saúde, Brasil, 2019.

Variável	Acesso geral	Acesso público dos atendidos no SUS	Acesso público dos não atendidos no SUS
		
	n (%)	n (%)	n (%)
Gênero			
Homem	6.985 (35,8)	3.526 (35,2)	2.261 (36,5)
Mulher	12.834 (64,2)	6.463 (64,8)	4.024 (63,5)
Cor/raça			
Branca	7.851 (47,4)	3.252 (40,4)	3.290 (59,1)
Negra	11.968 (52,6)	6.737 (59,6)	2.995 (40,9)
Nível socioeconômico			
Alto	10.160 (52,6)	4.015 (41,0)	4.471 (71,0)
Baixo	9.659 (47,4)	5.974 (59,0)	1.814 (29,0)
Território			
Sul	2.685 (15,6)	1.274 (15,8)	917 (14,3)
Sudeste	4.783 (47,2)	2.037 (42,4)	1.883 (54,0)
Centro-Oeste	2.220 (7,0)	970 (6,8)	839 (7,2)
Nordeste	6.617 (23,9)	3.672 (27,7)	1.811 (19,7)
Norte	3.514 (6,3)	2.036 (7,3)	835 (4,8)
Grupo interseccional			
Homens, brancos, alto nível socioeconômico	1.574 (10,0)	446 (6,2)	877 (15,9)
Homens, brancos, baixo nível socioeconômico	1.249 (7,2)	733 (8,3)	294 (5,4)
Homens, negros, alto nível socioeconômico	1.770 (8,1)	778 (7,0)	686 (9,6)
Homens, negros, baixo nível socioeconômico	2.392 (10,5)	1.569 (13,7)	404 (5,6)
Mulheres, brancas, alto nível socioeconômico	2.932 (17,7)	904 (11,6)	1.604 (27,5)
Mulheres, brancas, baixo nível socioeconômico	2.096 (12,6)	1.169 (14,3)	515 (10,3)
Mulheres, negras, alto nível socioeconômico	3.884 (16,8)	1.887 (16,3)	1.304 (18,0)
Mulheres, negras, baixo nível socioeconômico	3.922 (17,1)	2.503 (22,6)	601 (7,7)
Acesso aos medicamentos prescritos			
Não	3.139 (15,1)	6.906 (69,6)	6.124 (97,0)
Sim	16.680 (84,9)	3.083 (30,4)	161 (3,0)
Total	19.819	9.989	6.285

SUS: Sistema Único de Saúde.

Fonte: Elaborado pelos autores com base na análise de dados da PNS, 2019.

A prevalência de acesso geral encontrada neste estudo foi de 84,9% (Tabela 2). Quando se considerou apenas o acesso a medicamentos no SUS com prescrições originadas no próprio sistema, a prevalência observada foi de 30,4%. Já a prevalência de acesso público entre aqueles que tiveram prescrições originadas fora do SUS foi de 3,0%. Homens, participantes que referiram cor/raça branca, de alto nível socioeconômico e residentes da região Sul tiveram maiores prevalências de acesso geral. Quando analisado o acesso a medicamentos no setor público de prescrições originadas no SUS, maiores prevalências foram observadas em homens, respondentes de cor/raça negra, entrevistados de baixo nível socioeconômico e residentes da região Sudeste. No acesso público a medicamentos de prescrições originadas fora do SUS, a prevalência do desfecho entre homens e mulheres foi a mesma, enquanto maiores prevalências foram observadas em respondentes de cor/raça negra, entrevistados de baixo nível socioeconômico e residentes da região Nordeste.

**Tabela 2:** Prevalências observadas de acesso geral (público, privado e misto) e acesso público (via SUS) a medicamentos prescritos de respondentes atendidos ou não no SUS, de acordo com gênero, cor/raça, nível socioeconômico, território e grupo interseccional. Pesquisa Nacional de Saúde, Brasil, 2019.

Variável	Prevalência de acesso geral	Prevalência de acesso público dos atendidos no SUS	Prevalência de acesso público dos não atendidos no SUS
		
	% (IC95%)	% (IC95%)	% (IC95%)
Gênero			
Homem	86,8 (85,5–88,0)	30,7 (28,2–33,3)	3,0 (2,1–4,5)
Mulher	83,8 (82,7–84,8)	30,2 (28,3–32,2)	3,0 (2,2–4,0)
Cor/raça			
Branca	86,6 (85,2–87,8)	29,5 (26,9–32,1)	2,5 (1,8–3,4)
Negra	83,3 (82,1–84,5)	31,0 (29,0–33,0)	3,8 (2,7–5,4)
Nível socioeconômico			
Alto	86,6 (85,4–87,7)	28,6 (26,3–31,1)	2,3 (1,6–3,1)
Baixo	83,0 (81,7–84,2)	31,6 (29,5–33,7)	4,8 (3,3–7,1)
Território			
Sul	86,1 (84,1–87,9)	31,6 (28,2–35,3)	3,1 (1,8–5,3)
Sudeste	85,7 (84,1–87,2)	33,6 (30,6–36,7)	2,8 (1,9–3,9)
Centro-Oeste	85,5 (83,3–87,5)	26,3 (22,6–30,3)	1,8 (0,9–3,4)
Nordeste	83,3 (81,8–84,7)	26,5 (24,4–28,6)	4,2 (2,4–7,2)
Norte	80,5 (78,1–82,7)	27,7 (24,8–30,9)	2,5 (1,4–4,5)
Grupo interseccional			
Homens, brancos, alto nível socioeconômico	90,2 (87,7–92,2)	30,8 (23,8–38,8)	2,0 (1,0–4,0)
Homens, brancos, baixo nível socioeconômico	85,0 (81,8–87,7)	30,6 (25,7–36,1)	4,0 (1,8–8,9)
Homens, negros, alto nível socioeconômico	87,6 (84,9–89,9)	28,3 (23,5–33,6)	3,0 (1,3–6,6)
Homens, negros, baixo nível socioeconômico	84,1 (81,7–86,3)	31,9 (28,4–35,6)	5,1 (2,1–12,2)
Mulheres, brancas, alto nível socioeconômico	87,0 (84,9–88,9)	26,8 (22,3–31,8)	1,4 (0,9–2,2)
Mulheres, brancas, baixo nível socioeconômico	84,0 (81,6–86,2)	30,4 (26,4–34,6)	5,2 (3,2–8,5)
Mulheres, negras, alto nível socioeconômico	83,5 (81,5–85,4)	29,3 (26,2–32,6)	3,5 (2,0–6,0)
Mulheres, negras, baixo nível socioeconômico	80,6 (78,5–82,6)	32,5 (29,1–36,0)	4,7 (2,3–9,1)
Total	84,9 (84,0–85,7)	30,4 (28,8–32,0)	3,0 (2,3–3,9)

SUS: Sistema Único de Saúde. IC: intervalo de confiança.

Fonte: Elaborado pelos autores com base na análise de dados da PNS, 2019.

Homens brancos de alto nível socioeconômico tiveram a maior prevalência de acesso geral, enquanto mulheres negras de baixo nível socioeconômico tiveram a maior prevalência de acesso público dos atendidos no SUS. A maior prevalência de acesso público dos atendidos fora do SUS foi observada entre as mulheres brancas de baixo nível socioeconômico. A análise das prevalências dos desfechos de cada grupo interseccional, estratificado por macrorregião, também foi realizada. No entanto, as estimativas encontradas foram acompanhadas de baixa precisão, observada pelos amplos intervalos de confiança^a^.

A partir das análises nos modelos de regressão logística, observa-se na Tabela 3 que o OR de prevalência de acesso geral foi menor para as mulheres, respondentes de cor/raça negra, para aqueles de baixo nível socioeconômico (Modelos 1 e 2) e residentes da região Norte do país (Modelos 1, 2 e 3). Ao analisar os grupos interseccionais, verificou-se que, tanto na análise bivariada (Modelo 1), quanto na análise ajustada (Modelo 3), todos os grupos interseccionais, exceto homens negros de alto nível socioeconômico, tiveram menores OR de prevalência de acesso geral em relação aos homens brancos de alto nível socioeconômico.

**Tabela 3:** *Odds ratios* estimadas nos modelos de regressão logística para o acesso geral (público, privado e misto) a medicamentos prescritos de acordo com as variáveis de exposição. Pesquisa Nacional de Saúde, Brasil, 2019.

Variável	Modelo 1	Modelo 2	Modelo 3
		
	OR (IC95%)	OR (IC95%)	OR (IC95%)
Gênero			
Homem	1,00	1,00	-
Mulher	0,79 (0,70–0,89)	0,78 (0,69–0,89)	-
Cor/raça			
Branca	1,00	1,00	-
Negra	0,78 (0,68–0,89)	0,83 (0,72–0,96)	-
Nível socioeconômico			
Alto	1,00	1,00	-
Baixo	0,75 (0,66–0,86)	0,77 (0,68–0,87)	-
Território			
Sul	1,00	1,00	1,00
Sudeste	0,97 (0,79–1,19)	0,98 (0,80–1,21)	0,98 (0,80–1,20)
Centro-Oeste	0,95 (0,76–1,20)	1,01 (0,79–1,29)	1,01 (0,79–1,29)
Nordeste	0,81 (0,67–0,98)	0,89 (0,73–1,09)	0,89 (0,73–1,09)
Norte	0,67 (0,54–0,83)	0,73 (0,58–0,92)	0,73 (0,58–0,92)
Grupo interseccional			
Homens, brancos, alto nível socioeconômico	1,00	-	1,00
Homens, brancos, baixo nível socioeconômico	0,62 (0,44–0,87)	-	0,62 (0,44–0,87)
Homens, negros, alto nível socioeconômico	0,77 (0,55–1,08)	-	0,80 (0,57–1,14)
Homens, negros, baixo nível socioeconômico	0,58 (0,43–0,79)	-	0,60 (0,44–0,83)
Mulheres, brancas, alto nível socioeconômico	0,73 (0,55–0,98)	-	0,73 (0,55–0,98)
Mulheres, brancas, baixo nível socioeconômico	0,57 (0,42–0,77)	-	0,58 (0,43–0,78)
Mulheres, negras, alto nível socioeconômico	0,55 (0,42–0,73)	-	0,58 (0,44–0,77)
Mulheres, negras, baixo nível socioeconômico	0,45 (0,34–0,60)	-	0,48 (0,35–0,64)

OR: *Odds ratio*; IC: intervalo de confiança.

Fonte: Elaborado pelos autores com base na análise de dados da PNS, 2019.

Quanto ao acesso público dos atendidos no SUS (Tabela 4), entrevistados de baixo nível socioeconômico apresentaram maior OR de prevalência do desfecho quando comparado aos de alto nível socioeconômico (Modelo 2). A análise bivariada indicou que residentes da região Nordeste tiveram menor OR de prevalência desse tipo de acesso em relação aos moradores da região Sul (Modelo 1). Na análise ajustada, observou-se menor OR desse acesso também para os moradores das regiões Centro-Oeste e Norte (Modelos 2 e 3). Com relação à análise dos grupos interseccionais, não foram observadas iniquidades interseccionais para esse desfecho em nenhum dos modelos.

**Tabela 4:** *Odds ratios* estimadas nos modelos de regressão logística para o acesso público (via SUS) a medicamentos prescritos no SUS de acordo com as variáveis de exposição. Pesquisa Nacional de Saúde, Brasil, 2019.

Variável	Modelo 1	Modelo 2	Modelo 3
		
	OR (IC95%)	OR (IC95%)	OR (IC95%)
Gênero			
Homem	1,00	1,00	-
Mulher	0,98 (0,84–1,13)	0,99 (0,85–1,15)	-
Cor/raça			
Branca	1,00	1,00	-
Negra	1,07 (0,92–1,26)	1,17 (0,99–1,38)	-
Nível socioeconômico			
Alto	1,00	1,00	-
Baixo	1,15 (0,99–1,33)	1,17 (1,01–1,36)	-
Território			
Sul	1,00	1,00	1,00
Sudeste	1,09 (0,88–1,35)	1,07 (0,86–1,34)	1,08 (0,87–1,34)
Centro-Oeste	0,77 (0,59–1,00)	0,73 (0,56–0,96)	0,73 (0,56–0,96)
Nordeste	0,78 (0,64–0,95)	0,72 (0,59–0,89)	0,72 (0,59–0,89)
Norte	0,83 (0,66–1,04)	0,78 (0,61–0,99)	0,78 (0,61–0,99)
Grupo interseccional			
Homens, brancos, alto nível socioeconômico	1,00	-	1,00
Homens, brancos, baixo nível socioeconômico	0,99 (0,65–1,52)	-	1,03 (0,68–1,58)
Homens, negros, alto nível socioeconômico	0,88 (0,58–1,36)	-	0,96 (0,63–1,48)
Homens, negros, baixo nível socioeconômico	1,05 (0,71–1,55)	-	1,19 (0,80–1,75)
Mulheres, brancas, alto nível socioeconômico	0,82 (0,54–1,25)	-	0,83 (0,54–1,26)
Mulheres, brancas, baixo nível socioeconômico	0,98 (0,65–1,48)	-	1,01 (0,67–1,52)
Mulheres, negras, alto nível socioeconômico	0,93 (0,63–1,37)	-	1,05 (0,71–1,53)
Mulheres, negras, baixo nível socioeconômico	1,08 (0,73–1,60)	-	1,22 (0,83–1,81)

OR: *Odds ratio*; IC: intervalo de confiança. SUS: Sistema Único de Saúde.

Fonte: Elaborado pelos autores com base na análise de dados da PNS, 2019.

Em relação ao acesso público dos atendidos fora do SUS (Tabela 5), maior OR de prevalência do desfecho também foi encontrado entre respondentes de baixo nível socioeconômico, quando comparado aos de alto nível socioeconômico (Modelo 1 e 2). Tanto na análise bivariada (Modelo 1), quanto na análise ajustada (Modelo 3), o grupo interseccional de mulheres brancas de baixo nível socioeconômico teve maiores OR de prevalência desse tipo de acesso em relação aos homens brancos de alto nível socioeconômico. No entanto, os achados mencionados foram acompanhados de intervalos de confiança amplos, devido ao tamanho amostral reduzido em relação aos outros desfechos. Um quarto modelo – não apresentado nas tabelas – foi estimado para testar a interação entre as variáveis território e grupos interseccionais para os três desfechos. Contudo, nenhuma interação expressiva foi identificada.

**Tabela 5:** *Odds ratios* estimadas nos modelos de regressão logística para o acesso público (via SUS) a medicamentos prescritos fora do SUS de acordo com as variáveis de exposição. Pesquisa Nacional de Saúde, Brasil, 2019.

Variável	Modelo 1	Modelo 2	Modelo 3
		
	OR (IC95%)	OR (IC95%)	OR (IC95%)
Gênero			
Homem	1,00	1,00	-
Mulher	0,98 (0,62–1,55)	1,01 (0,63–1,62)	-
Cor/raça			
Branca	1,00	1,00	-
Negra	1,57 (0,98–2,50)	1,51 (0,89–2,56)	-
Nível socioeconômico			
Alto	1,00	1,00	-
Baixo	2,18 (1,29–3,67)	2,10 (1,24–3,56)	-
Território			
Sul	1,00	1,00	1,00
Sudeste	0,88 (0,46–1,70)	0,81 (0,43–1,52)	0,81 (0,43–1,54)
Centro-Oeste	0,56 (0,24–1,33)	0,48 (0,20–1,14)	0,49 (0,20–1,16)
Nordeste	1,35 (0,61–3,01)	1,07 (0,46–2,49)	1,09 (0,47–2,52)
Norte	0,79 (0,35–1,79)	0,60 (0,25–1,41)	0,62 (0,26–1,45)
Grupo interseccional			
Homens, brancos, alto nível socioeconômico	1,00	-	1,00
Homens, brancos, baixo nível socioeconômico	2,03 (0,68–6,08)	-	2,04 (0,68–6,12)
Homens, negros, alto nível socioeconômico	1,50 (0,51–4,44)	-	1,50 (0,49–4,57)
Homens, negros, baixo nível socioeconômico	2,63 (0,82–8,49)	-	2,63 (0,89–7,75)
Mulheres, brancas, alto nível socioeconômico	0,68 (0,31–1,50)	-	0,69 (0,31–1,51)
Mulheres, brancas, baixo nível socioeconômico	2,67 (1,12–6,36)	-	2,64 (1,12–6,25)
Mulheres, negras, alto nível socioeconômico	1,74 (0,71–4,27)	-	1,77 (0,71–4,41)
Mulheres, negras, baixo nível socioeconômico	2,37 (0,84–6,67)	-	2,37 (0,81–6,98)

OR: *Odds ratio*; IC: intervalo de confiança. SUS: Sistema Único de Saúde.

Fonte: Elaborado pelos autores com base na análise de dados da PNS, 2019.

## DISCUSSÃO

Ecoando outros estudos de base populacional, a prevalência de acesso geral a medicamentos prescritos encontrada foi alta, quando considerado qualquer tipo de fonte de obtenção (público, privado e misto)^12,14^. Verificou-se maior prevalência de acesso geral em segmentos populacionais de maior privilégio, como homens brancos de alto nível socioeconômico. Quando se considera apenas o acesso a medicamentos no sistema público de prescrições originadas no próprio sistema, a prevalência é baixa, mas o cenário é revertido para maior prevalência de acesso entre pessoas de cor/raça negra e de baixo nível socioeconômico. Inclusive, observou-se maior prevalência de acesso público dos atendidos no SUS para mulheres negras de baixo nível socioeconômico. Isso indica que o SUS é uma importante fonte de acesso para aqueles que não conseguem obter os medicamentos fora do sistema público^29^. A modalidade de copagamento, que é adotada por outros sistemas universais de saúde como principal forma de obtenção de medicamentos, é considerada um dificultador do acesso. Maiores valores de copagamento estão associados a uma redução no volume de dispensação de medicamentos, principalmente para os mais pobres, o que leva à descontinuação do tratamento, comprometendo a eficácia dos cuidados de saúde prestados^9^. Nossos achados corroboram outros estudos que indicam que a provisão gratuita de medicamentos diminui as iniquidades de acesso^11,13,14,15,16,24^, apontando ainda que o acesso a medicamentos por meio do SUS é um importante instrumento de combate às iniquidades interseccionais.

Apesar disso, a baixa prevalência de acesso identificada para a obtenção no SUS de medicamentos prescritos no próprio sistema é preocupante, principalmente pelos indicativos de queda nos últimos anos. Na análise dos dados obtidos a partir da Pesquisa Nacional por Amostra de Domicílios de 2008, Boing et al.^11^ identificaram prevalência de acesso público de 45,3%, enquanto que, na PNS de 2013, o percentual identificado foi de 31,6%^23^. Quanto ao território, representado pelas macrorregiões do país, a análise dos da PNS de 2019 evidenciou que as iniquidades regionais de acesso público a medicamentos persistem. Drummond et al.^14^, ao analisarem a PNS de 2013, também identificaram maiores prevalências de acesso a medicamentos prescritos nas regiões mais desenvolvidas e com maior densidade populacional. Esses achados apontam que, apesar dos avanços obtidos com a implementação de políticas de medicamentos e de AF no país, a provisão de medicamentos pelo sistema público de saúde ainda permanece como um desafio importante^30^.

Além disso, a presença simultânea de um sistema público universal no Brasil e um crescente processo de privatização do acesso aos cuidados de saúde, intensificado ainda pelo subfinanciamento público da saúde, também são preocupantes^31^. Neste estudo, observou-se que uma parcela significativa da população que não teve suas necessidades supridas pelo SUS tentou obter os medicamentos prescritos por meio do sistema público de saúde. Para ela, a prevalência de acesso aos medicamentos prescritos foi ainda mais baixa. Além das já conhecidas fragilidades na estruturação dos serviços farmacêuticos no país, como a disponibilidade dos medicamentos nas farmácias do SUS^12^, tem-se o desconhecimento dos prescritores do setor privado das relações de medicamentos essenciais, listas de referência que devem nortear a oferta, a prescrição e a dispensação de medicamentos no SUS, e a não adesão a essas listas por parte dos prescritores do setor público. Ao observar as demandas de acesso a medicamentos pela via judicial em diferentes estados brasileiros, verifica-se que a maior parte das prescrições são originadas fora do SUS e em aproximadamente 75% dos casos há alternativa terapêutica ao medicamento prescrito disponível pelo SUS^34,35^.

Diferentemente do acesso geral, o acesso público não foi marcado por iniquidades interseccionais de expressiva magnitude. Nos modelos testados para o acesso público dos atendidos no SUS, não foram identificadas diferenças significativas entre os grupos interseccionais. Observa-se que a amplitude dessa medida entre os grupos interseccionais para o acesso geral (90,2% a 80,6%) é maior quando comparada ao acesso público dos atendidos no SUS (32,5% a 26,8%). Isso sugere que as diferenças de acesso observadas entre os segmentos populacionais são minimizadas quando o medicamento é prescrito e obtido no sistema público de saúde. Monteiro et al.^36^, ao avaliarem a Política de Medicamentos Genéricos, também observaram não haver diferença estatisticamente significativa na utilização de medicamentos genéricos na população da cidade de São Paulo de acordo com idade, sexo e escolaridade.

A análise interseccional empregada favoreceu a identificação de grupos populacionais mais invisibilizados. Além disso, permitiu observar que, enquanto as mulheres tiveram OR de acesso geral aproximadamente 20% menor, as mulheres brancas de alto nível socioeconômico tiveram OR 27% menor e as mulheres negras de baixo nível socioeconômico, OR 55% menor, quando comparadas aos homens brancos de alto nível socioeconômico. A pesquisa de Katrein et al.^17^, ao analisar a prevalência de acesso a medicamentos para o tratamento de doenças crônicas, já havia indicado situação de maior vulnerabilidade entre as que têm maior número de doenças e as que são mais pobres.

Ainda que importantes desafios estejam colocados no emprego de uma perspectiva interseccional em estudos epidemiológicos^37^, destaca-se tal abordagem como ponto forte deste estudo. Inexistem na literatura outros trabalhos na perspectiva da interseccionalidade que sejam comparáveis a este. Os estudos até então realizados, que demonstraram a existência de iniquidades no acesso a medicamentos no Brasil^17,18,24^, trabalharam as variáveis como gênero, cor/raça e escolaridade de forma independente e consideraram que cada uma delas tem um efeito isolado.

Recentes revisões demonstraram que a operacionalização das variáveis que caracterizam as posições sociais pode ocorrer de diferentes formas^25,26^. Neste estudo, assim como na maior parte daqueles analisados nas revisões mencionadas, operacionalizou-se a interseccionalidade utilizando-se uma abordagem exclusivamente intercategórica, a partir da combinação de categorias de interesse. No entanto, as comparações entre grupos, por si só, podem fornecer uma visão limitada das iniquidades em saúde. Além de desconsiderar a heterogeneidade intracategórica, esse tipo de análise focaliza as categorias sociais de nível individual, sem considerar o efeito de contextos sociais mais amplos^38^.

Dentre as limitações do estudo, destaca-se também a inclusão de somente pessoas atendidas por serviço de saúde nas duas últimas semanas anteriores à entrevista. Considerando que o acesso a medicamentos prescritos está diretamente relacionado com o acesso aos cuidados em saúde, são excluídas as pessoas que não chegam aos serviços de saúde. Observou-se que pessoas negras e de baixo nível socioeconômico possuem maiores chances de relatar dificuldade de acesso aos serviços de saúde^39^. Ademais, pode-se assumir um possível viés de memória em decorrência do período recordatório e autorrelato sobre os desfechos analisados. No entanto, estudo que comparou períodos recordatórios diferentes identificou pouco viés nas prevalências observadas, recomendando-se a adoção de 14 dias como período recordatório para permitir comparações entre estudos^40^.

Conclui-se que a prevalência de acesso geral a medicamentos prescritos é maior para os segmentos populacionais de *status* social mais alto, enquanto o acesso público, que ainda é muito pequeno, favorece os de *status* social mais baixo, inclusive, ao se considerar a intersecção de múltiplos eixos de marginalização. Evidenciou-se, portanto, que o acesso a medicamentos pelo SUS está relacionado com menores iniquidades sociais.

## Footnotes

a Material Suplementar disponível em: https://doi.org/10.7910/DVN/I0CL2L
